# The Safety of an Adjuvanted Autologous Cancer Vaccine Platform in Canine Cancer Patients

**DOI:** 10.3390/vetsci5040087

**Published:** 2018-10-12

**Authors:** Chris Weir, Annika Oksa, Jennifer Millar, Miles Alexander, Nicola Kynoch, Zoe Walton-Weitz, Peter Mackenzie-Wood, Felicia Tam, Hope Richards, Richard Naylor, Katrina Cheng, Peter Bennett, Nikolai Petrovsky, Rachel Allavena

**Affiliations:** 1 Northern Blood Research Laboratory, Kolling Institute of Medical Research, Royal North Shore Hospital and the Sydney Medical School, University of Sydney, Sydney NSW 2065, Australia; Chris.weir@sydney.edu.au; 2 School of Veterinary Science, The University of Queensland, Gatton Campus, Gatton, QLD 4343 Australia; a.oksawalker@uq.edu.au (A.O.); r.allavena@uq.edu.au (R.A.); 3 Willougby Veterinary Hospital, Sydney, NSW 2068, Australia; jmil2396@uni.sydney.edu.au (J.M.); nicola.kynoch@gmail.com (N.K.); zoewaltonweitz@gmail.com (Z.W.-W.); northvet@bigpond.net.au (P.M.-W.); jandhpitman@gmail.com (H.R.); 4 Elanora Veterinary Hospital, Sydney, NSW 2101 Australia; kerri.and.miles@gmail.com; 5 Castle Hill Veterinary Hospital, Sydney, NSW 2154, Australia; felicia.cht@gmail.com (F.T.); clinic@castlehillvets.com.au (R.N.); 6 Sydney School of Veterinary Science, Faculty of Science, University of Sydney, NSW 2006, Australia; Katrina.cheng@sydney.edu.au (K.C.); peter.bennett@sydney.edu.au (P.B.); 7 Flinders University Bedford Park, Adelaide, SA 5042, Australia; nikolai.petrovsky@flinders.edu.au; 8 Vaxine Pty Ltd., Bedford Park, Adelaide, SA 5042, Australia

**Keywords:** vaccine, autologous, canine, adjuvant, Advax™

## Abstract

Canine cancer rates are similar to humans, though the therapeutic options might be limited. Inducing a patient’s own immune system to have an anti-tumor response is an attractive approach to cancer therapy. In this safety study, autologous tumor vaccines produced specifically for each canine patient were combined with Advax™, a novel non-inflammatory immunomodulator and vaccine adjuvant and were tested for safety in a diverse range of patient presentations alone or in combination with other treatments. Canine patients had their tumor biopsied, debulked or resected and the tumor antigens were processed into an autologous vaccine formulated with Advax™ adjuvant with or without rhizavidin as an additional immune stimulant. Patients treated early in the trial received two intramuscular (IM) doses, 2 weeks apart. As the study progressed and no issues of safety were observed, the protocol was changed to weekly vaccinations for 4 weeks followed by monthly booster shots. Over the 150 I.M injections delivered to date, the vaccine was found to be very safe and no significant adverse reactions were observed. These results justify ongoing development and future controlled studies of this autologous vaccine approach.

## 1. Introduction

Canine malignancy rates are similar to those of humans, however, canine cancer patients can have limited therapeutic options due to financial access or safety barriers. Cancer immunotherapy is a rapidly growing area of human oncology [1], which may also have benefits for canine cancer patients. Furthermore, recent One Health initiatives have highlighted the use of pet dogs with spontaneous cancer as translational models for the development of human cancer therapies [2,3,4]. While rodent models provide a rapid and relatively inexpensive approach to studying cancer progression and new therapies, they have significant limitations [2,4]. Rodent oncology models are typically induced with the potential to create significant divergence in the immune profile of the tumor microenvironment from the spontaneous cancers seen in dogs and humans. Further, human and canine cancers are polygenic, but rodent models are usually driven by select causal genetic mutations [2]. Using canine patients for testing of potential immunotherapies has other key advantages such as accessibility, shared environmental exposure of humans, phenotypic diversity, similar patient size and closer genetic similarity to humans [5]. Spontaneous canine cancers reflect the phenotypic and genetic diversity of the approximately 400 breeds, thereby having similarities to an outbred human population [6]. The strong breed association of certain cancers makes diagnosis and prediction of the clinical course of canine cancer easier in these cases, with extensive published data on expected survival times relative to the grading and staging of various cancers [7] Like humans, dogs tend to develop cancer from middle age, with the majority of dogs presenting at >6 years of age, equivalent of 60–95 human years, which is when most malignancies present in humans [8]. Further, greater than 50% of dogs over 10 years of age will develop cancer [2]. Therefore, with large populations in developed countries, pet dogs represent a substantial translational resource for cancer therapy development [2]. Differences lie in canine patients generally presenting with more advanced disease than human cancers, and minimal current access to antibody-based immunotherapies due to species differences. Depending on the owner’s wishes and budget, treatment options include palliation, surgery, chemotherapy, radiotherapy or a combination. Overall, canine patients tend not to be treated as aggressively or consistently as humans, and euthanasia of patients is used extensively in veterinary medicine, leading to shorter survival times. As canine cancer progression tends to be more rapid than in people, this can lead to significantly shorter duration of canine cancer trials. This combined with the strong parallels between humans and dog cancers is a major advantage as it allows for new cancer therapies to be assessed rapidly in dogs and then translated to the clinic for the benefit of both humans and dogs.

Stimulating a patient’s own immune system to target and attack the neoplastic cells has become a new weapon in the fight against cancer, as with humans, canine cancers contain a unique set of mutated tumor proteins enabling a patient’s own cancer-derived proteins to be used to produce a vaccine [9]. The advantage of an autologous vaccine is that MHC molecules as well as tumor-associated antigens are patient specific [10]. However, depending on the cancer type, tumor-specific antigens may have a low level of immunogenicity, or the tumor may have a low percentage of unique mutated proteins [11]. Bacterial products can be used to increase the level of immunogenicity to tumor antigens, for example, immune stimulants such as Bacillus Calmette-Guerin (BCG) have been used with irradiated autologous tumor cells in bladder cancer and other tumor types [12,13].

Several autologous cancer vaccines that showed promise in early phase studies have failed to deliver survival benefits for patients with advanced disease, showing benefits only for early stage disease [14,15]. With the advent of immune checkpoint inhibitors (CPI) which overcome tumor immune suppression in certain cancer types [16], the concept of combining CPI with autologous tumor vaccines to improve efficacy is now being explored using a variety of vaccine preparations [17,18]. One downside to the use of bacterial immune stimulants such as BCG or Freund’s adjuvant in cancer therapy is their inflammation-associated side effects, with inflammation in turn upregulating immune checkpoint receptors such as PD-1 on T cells, resulting in immune suppression [19]. Hence, we hypothesized that the use of a non-inflammatory adjuvant may provide an enhanced safety profile but also avoid induction of regulatory T cells that suppress anti-tumor responses. Advax™ is a unique non-inflammatory adjuvant produced from delta isoform of inulin, a natural plant polysaccharide. Delta inulin adjuvants have been shown to induce balanced immune responses encompassing both humoral and cellular immunity [20]. This has been shown to translate to enhanced vaccine protection against a broad range of pathogens including Japanese encephalitis virus [21,22], West Nile virus [23], hepatitis B virus [24], influenza [25], HIV [26], SARS [27], coronavirus [27], Listeria monocytogenes [28] and Bacillus anthracis [29], as well as non-infectious diseases such as Alzheimer’s disease [30]. Advax™ induces minimal inflammation even when compared to alum adjuvant formulations [31] and has been shown to be safe and effective in human trials of vaccines against influenza [32] hepatitis B [31] and allergy [33]. Therefore, we sought to conduct a Phase 1 safety trial in canine cancer patients of our autologous cancer vaccine containing Advax™ adjuvant with or without an additional bacterial protein immune stimulant rhizavidin [34] to generate safety and proof of concept data for this novel oncology approach. We hypothesized based on our own previous cancer vaccine development work [35], that using streptavidin as an immune stimulant with the addition of a similar foreign bacterial protein such as rhizavidin may improve the immune response to vaccination. 

## 2. Materials and Methods 

### 2.1. Study Outline and Aims

The aims of this study were to assess the safety of the autologous vaccine approach. Secondary aims were to assess for subjective responses and decide what tumour types the vaccine might best be targeted at in future studies. We also wished to establish whether the addition of rhizavidin as an additional immune stimulant to the vaccine was safe. Alternations to dosing frequency and timing were explored during the study, as ongoing murine studies revealed greater efficacy of more frequent vaccine dosing. Nevertheless, timing of vaccine doses was frequently dictated by the ability of owners to bring animals to the vet clinics.

Approval was obtained from the Animal Care and Ethics Committee of the University of Queensland; (certificates ANRFA/SVS/346/15; SVS/328/14/JMKT) to undertake a Phase 1 safety trial of the autologous vaccine approach in dogs presenting with various cancers. Following the provision of vaccine information, owners gave informed written consent to include their dog in the study. Participation was irrespective of tumor type, prior cancer treatment or age of the dog. Inclusion criteria included an animal with a tumor that could be sampled or removed to provide enough material for a vaccine to be produced and where the owners were prepared to give consent or participate in the study. Exclusion criteria included a tumor that could not be sampled; the patient had systemic illness that could impact vaccine response (such as marked fever, immune suppression, organ failure) or were not expected to live long enough for the patient to benefit from the vaccination (<1 month). The trial cessation criteria were any significant side effects requiring hospitalization (anaphylactic reaction, moderate to severe gastroenteritis, other systemic inflammatory reactions) in up to three animals or vaccination causing death in any animal.

Additional patients at three Sydney (Australia) veterinarian practices were treated on compassionate grounds after owners gave informed consent. All vaccines were provided at no cost to the owners. 

**Figure 1:**
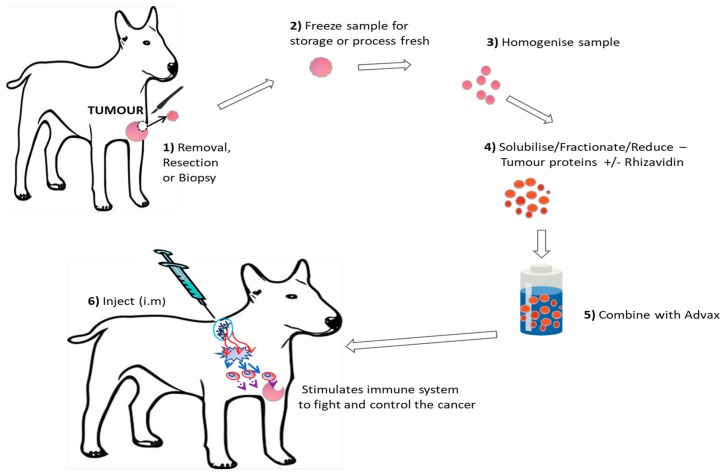
Visual Representation of the autologous vaccine production process.

Figure 1 demonstrates the vaccine production process.

### 2.2. Vaccine Production

This autologous vaccine production protocol was tested and found to be safe in murine cancer models prior to starting this trial. Biopsied, resected or debulked tumor samples obtained from each individual patient were processed fresh or stored frozen at −20 °C until processed at laboratories at the University of Queensland or the University of Sydney. A minimum of 0.1 g sample was required and each sample was processed into the vaccine which produces four doses via the following method. For each 0.1 g of tumor, 1 mL of tumor buffer (50 mM Hepes, 0.15 M NaCl, 0.01% SDS pH 7.2) was added. The Tumor sample was then homogenized (Ultraturrax) to obtain a uniform suspension with no fragments of tumour remaining and then spun at 15,000 rpm for 5 min and the soluble lysate was collected. Soluble lysate contained between 5 to 30 mg of material per mL depending on the tumour type and origin. Dithiothreitol (DTT) was then added to a final concentration of 0.1 mM and 10 mg Advax™ was added per dose. Each final vaccine dose contained 0.2 mL Advax™ at 50 mg/mL with 0.2 mL of tumour lysate with or without 25 µg of rhizavidin and 0.01 mg/mL Thiomersal as a preservative. Prepared vaccine was stored at 4 °C until use. Murine studies later demonstrated no additional efficacy from the addition of the immune stimulant rhizavidin. To test if this was also the case for spontaneous canine tumors, approximately 50% of dogs received rhizavidin in their vaccine to compare safety and preliminary clinical outcomes. The rhizavidin used in this study was donated by Dr George Anderson (Naval Research Laboratory, Washington, DC, USA) and had an endotoxin level of less than 0.1 EU/mg.

### 2.3. Vaccine Administration

Autologous vaccine was injected intramuscularly (I.M) at a volume of 0.4 mL at rotating sites as distal to the original tumor location as possible. Initially, patients received two doses of vaccine 2 weeks apart but as more preclinical safety and efficacy data were generated, the regimen was switched to an induction phase of four vaccine doses at weekly internals and then monthly boosts thereafter. Some animals received (Table 1) a more intense weekly or monthly protocol based on the aggressiveness of the cancer and the availability of owners to come to the clinic.

### 2.4. Histopathology and Tumor Grading

For inclusion into the study, owners were requested to have histopathology and any applicable tumor grading performed on their dog’s tumor. This was done by a veterinary pathologist at a commercial pathology laboratory (IDEXX, Laboratories, Australia) or via in-house assessment at the University of Queensland by veterinary pathologists. Standard four, micron paraffin-embedded formalin fixed sections stained with Haematoxylin and Eosin were assessed. When applicable, Toluidine Blue was used to identify mast cells, and Masson’s Trichrome to examine connective tissues. Immunohistochemistry for T- and B-cells was performed on all canine lymphoma samples to allow for subtyping the lymphoma using the WHO classification system [36]. Canine mast cell tumors were graded as high or low according to guidelines of Kiupel et al. (2011) [37]. Canine mammary tumors were classified according to Goldschmidt et al. (2011) [38].

### 2.5. Patient Status

At each clinical review, the dogs involved in the trial received a complete physical examination, and tumor measurement, shape and appearance were recorded in the patient’s clinical record and were documented by digital photography. Patients with measurable residual disease had their clinical responses recorded according to the Veterinary Cooperative Oncology Group consensus document for solid tumors [39]. The following classification was used to characterize clinical response: Complete Response (CR) for the disappearance of all target lesions; Partial Response (PR) for at least 30% reduction in the sum of diameters of target lesions; Progressive Disease (PD) for either the appearance of one or more new lesions or at least a 20% increase in the sum of diameters of target lesions; Stable Disease (SD) for less than 30% reduction (PR) or 20% increase (PD) in the sum of diameters of target lesions. All palpable lymph nodes were measured and recorded, and if >10 mm in size, a fine needle aspirate was performed to evaluate for metastatic disease in the enlarged node.

At the initial visit, whenever possible, the dogs received complete staging of their disease, including a complete blood count, a complete biochemistry panel, urinalysis, and diagnostic imaging to determine the presence of metastases. The amount of staging performed depended on the veterinary facility the dog was presented to, cost associated with testing, and the cancer type. For example, dogs that presented with mast cell tumors had abdominal ultrasounds performed whenever possible, and dogs that presented with carcinomas or sarcomas had thoracic radiography performed, as these locations represent the metastatic pattern for that particular cancer. The cancers were staged using the information received from these tests. 

During the vaccination appointments, in addition to a physical examinations, dogs were monitored for any adverse events post IM injection. Heart rate, respiratory rate, and temperature were monitored and recorded for a 2-h period post vaccination. The vaccine injection site was observed for any signs of swelling, redness, or ulceration. Any adverse events observed were reported according to the Veterinary Cooperative Oncology Group–Common Criteria for Adverse Events (VCOG–CTCAE) [40].

## 3. Results

### 3.1. Treatment of Advanced Cancers with Autologous Tumor Vaccine

Twenty-seven pet dogs (19 male and 8 female) of a variety of pure and mixed breeds presented with 11 different types of cancer. They were all treated with vaccines prepared from their own tumor as part of this pilot Phase 1 safety study. 

Of dogs included in the intervention arm of the study, the majority had a significant burden of disease at vaccination. This included inoperable tumors, relapse following other therapies, metastases or residual tumor remaining after surgery. All 27 dogs enrolled in the active study were followed up at the census date (June 2018) and the following assessments were given: Dead/euthanased, Stable disease (SD), Complete Remission (CR), New Cancer (NC: new cancer of different histiogenesis from primary treated cancer). Two of the 27 patients were lost to follow up (LTFU) due to owners moving. Table 1 outlines the clinical features of the 27 dogs enrolled in the study.

### 3.2. Vaccine Safety

With over 150 vaccine doses administered, there were no major adverse events recorded. There was no evidence of local injection site reactions (swelling, erythema, nodules, ulcers), or systemic reactions (fever, lethargy) in response to the vaccine. No cases of anaphylaxis were recorded. Only two grade 1 reactions were observed, as classified by the VCOG-CTCAE. These two grade 1 events were in dogs already suffering from osteoarthritis that were reported by their owners to be more lame in the 24 h period post vaccination. Rhizavidin, which in preclinical trials was shown to be safe but did not increase efficacy (unpublished data), was incorporated into the vaccine for some patients (n = 13) as an additional immune stimulant. A similar number of patients (n = 12) received vaccine without rhizavidin to provide a comparison. No significant adverse events were recorded in dogs receiving vaccine with or without rhizavidin, matching preclinical findings. Increasing dosing schedule and frequency did not result in any dosing-related adverse events.

### 3.3. Vaccine Efficacy

Many of the dogs enrolled in this Phase 1 trial were also receiving or had received other cancer therapies, and this plus the diversity of tumors and breeds of dogs, meant that any additional impact of the vaccine on the natural history of their cancers cannot be determined from this safety trial. However, 11 dogs had no progression of their tumor (Stable Disease), and one dog went into complete remission (CR) after treatment with the vaccine alone. Two dogs diagnosed with a high grade mast cell tumor exceeded their expected survival by more than 5 months at the consensus date (9 month survival at census, versus 4 months expected [41]). The one dog with complete remission had a diagnosis of non-epitheliotrophic T-cell lymphoma, with resolution of the 13 lesions present at the time of initial enrollment in the trial. The data suggested better than expected outcomes in some immunized patients although the study was not designed for analysis of efficacy. This suggestion of clinical benefit, particularly in those dogs receiving the Advax™ vaccine without addition of rhizavidin on a weekly induction schedule followed by monthly boosters, supports further testing in placebo-controlled clinical trials.

### 3.4. Vaccine Composition

**Figure 2:**
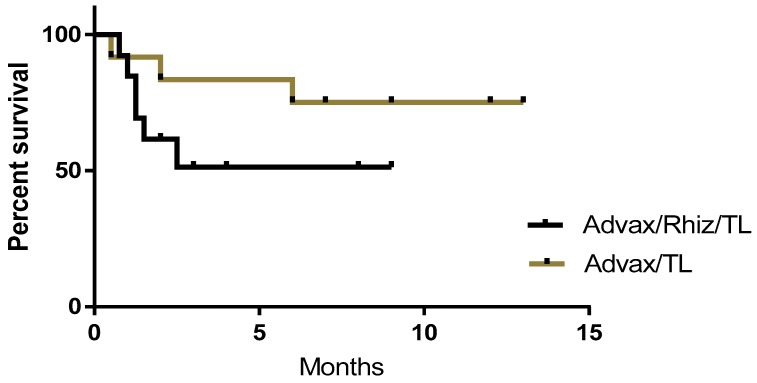
Survival curves comparing canine cancer patients vaccinated with autologous tumor lysate (TL) plus Advax™ adjuvant with or without rhizavidin (Rhiz). While the difference between groups is not statistically significant, there is a clear trend for the vaccine containing rhizavidin to be associated with shorter survival times.

Some versions of the vaccine included rhizavidin as an additional immune stimulant in combination with tumor protein and Advax™ adjuvant. These dogs were vaccinated with tumor protein plus rhizavidin (25 µg) and Advax™ (10 mg) to a total volume of 0.4 mL (n = 13). Subsequently, as no benefit was demonstrated in mouse models from the addition of rhizavidin, patients entering the study in Sydney received vaccine formulations without rhizavidin. At the time of the census cutoff, the current status and survival period post-vaccination of each patient receiving vaccinations with or without rhizavidin were recorded (Figure 2). While not statistically significant, a higher proportion of dogs were alive at the census in patients that received vaccine without (75%), compared to with (51%) rhizavidin 

### 3.5. Dose Schedule

**Figure 3:**
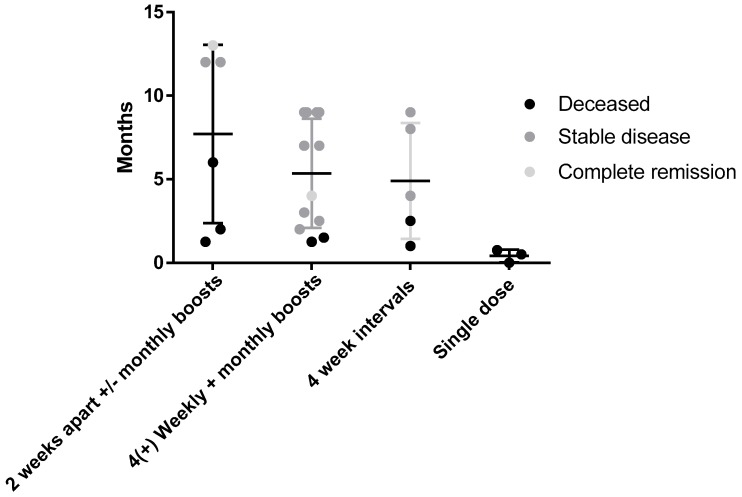
Survival comparison and outcome of different vaccine dosing schedules. No significant difference was seen between groups receiving either the weekly 4× with monthly boosts (n = 12) and monthly interval (n = 5) groups with 83% and 60% of patients being alive, respectively, at the time of the census. This compares to 50% in the second weekly group and 0% in groups that received a single dose (indicative of end stage terminal disease).

One of the challenges of translating from preclinical rodent models of cancer to treating canine patients is establishing the most effective dosing schedule. Regimens of two doses 2 or 4 weeks apart were effective in two murine models of cancer, so initially dogs were treated using this same schedule followed by monthly boosts. After seeing no adverse events in initial subjects and with new preclinical data confirming better efficacy with weekly vaccinations in murine models, this schedule was adopted for subsequent canine patients. Figure 3 shows the outcome of patients receiving different vaccine schedules regardless of tumor type.

### 3.6. Surgery and Response

**Figure 4:**
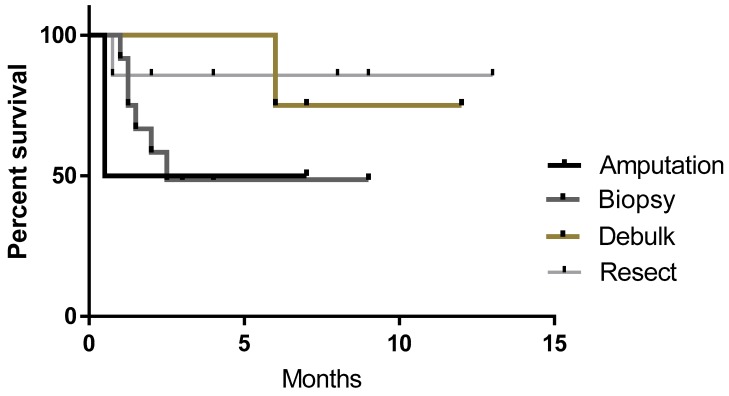
Survival curves of patients receiving biopsy, resection, debulking or amputation to collect sample. No significant difference was seen between groups, however, survival rates were 50% for amputation, 49% for biopsy, 75% for debulking and 86% for resectable tumors at the time of the census.

Patients enrolled in this study had tumor tissue harvested via complete or incomplete resection, debulking of primary tumor, biopsy or in the case of two dogs with osteosarcoma, amputation and sample collection. Figure 4 shows the survival curves of the four different types of sample collection. Resectable tumors (n = 7) had the highest rate of survival (86%) at the time of the census compared to debulking (n = 4) 75%, amputation (n = 2) 50% and biopsy (n = 12).

### 3.7. Response in Different Tumor Types

**Figure 5:**
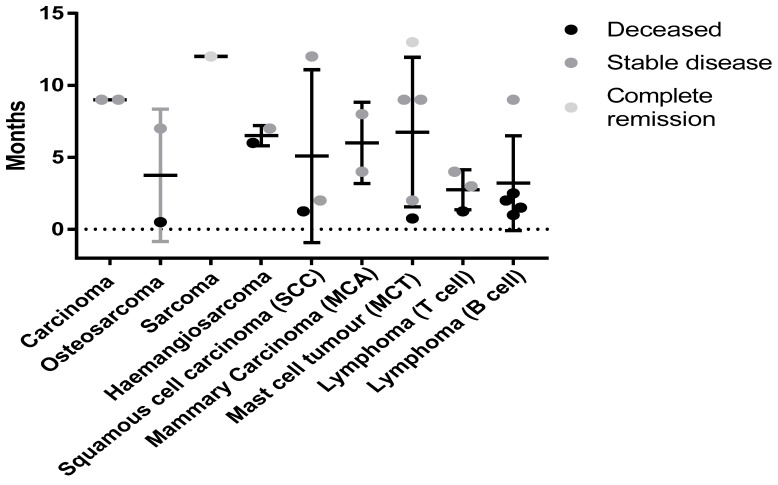
Status of canine patients with different neoplasms at the time of census. No significant difference was seen due to low numbers for each cancer type. Tumor types such as carcinomas (MCA and typical) had 100% survival with all patients having stable disease at the time of the census. Mast cell Tumors (MCT) may also be responding to this therapy with 80% of patients alive and one in complete remission (12 months from initial treatment) at the time of the census.

While this study was primarily designed to demonstrate the safety of the autologous vaccine formulated with Advax™ adjuvant, the data might provide initial insights into which tumor types may respond best to this vaccine approach. A variety of both solid and blood tumors were treated. Figure 5 shows the survival times and disease/patient status for the different tumor types.

## 4. Discussion

While traditional laboratory rodent models of cancer are useful for the initial evaluation of vaccine preparations, dogs provide a clinical presentation and scenario better matching that of human patients [2,3,4]. Dogs naturally develop similar cancers to humans, with correlating histopathological appearance, metastatic pattern, and response to therapy [42]. In addition, equivalent cancers are associated with similar genetic mutations in dogs and humans [42].

In this Phase I safety trial, no adverse reactions were observed when the autologous cancer vaccine containing Advax™ was administered alone or in combination with a variety of other cancer treatments. These results confirm the safety of this autologous vaccine approach for the treatment of canine cancers. Neither the vaccine when given alone or when combined with standard of care (SOC) therapy was associated with any pattern of adverse events, suggesting the vaccine is safe to use as a stand-alone therapy or combined with SOC.

While only designed to assess safety across a wide range of tumour types, some anecdotal suggestions of possible efficacy in some subjects were reported by treating clinicians, despite the canine patients presenting in various stages of disease (curable via surgical excision versus end-stage metastatic disease) and a variety of tumor types. Vaccinated dogs with residual or metastatic disease often survived longer than published predicted survival times, suggesting the vaccine had a positive clinical benefit, but more patients are needed to allow for assessment of statistical significance. Vaccination also appeared anecdotally to stop or at least slow the reoccurrence of tumors after resection, especially in patients with mast cell tumors. At the time of writing, one dog with non-epitheliotrophic T-cell lymphoma was in complete remission, with the disappearance of all lesions recorded pre-vaccination. This dog received no other treatments in conjunction to the autologous vaccine, and at the census date had no visible lesions. Although the efficacy data provided is preliminary, the autologous vaccine produced positive clinical benefits including remissions, static disease and amelioration of cancer-associated symptoms, with some animals exceeding the predicted survival times for their tumor and stage.

The ability to produce an autologous vaccine with a lag time of only a few hours between surgery and treatment, highlights its applicability to clinical situations. Furthermore, fresh tumor samples once frozen can be stored indefinitely until the vaccine is required if using in an adjunct setting. Further, many dog owners do not pursue current best practice cancer therapies such as radiotherapy or chemotherapy for their pets due to financial considerations. Likewise, many owners are very concerned about cytotoxic urine and faeces near or around their home associated with chemotherapy, and dog owners with young families often cite this as a reason for declining chemotherapy. Immunotherapy or immunization has no such associated hazards. 

At the time of census, survival rates of 50% for amputation, 49% for biopsy, 75% for debulking and 86% for resectable tumors were observed. The results indicate complete resection or debulking of tumor likely improves outcome as less residual disease will be present and this gives more time for vaccinations to stimulate the immune system. Further, with complete resection, surgical cure may have been achieved by the surgery alone. While biopsied subjects had a reduced response rate, this is most likely due to higher residual disease burden; although some longer term survivors (n = 3) with stable disease indicate biopsy samples and vaccination as an approach still demonstrated a positive clinical benefit.

While there was no significant difference in survival between dose schedules at the time of the census, of patients receiving four weekly doses, then monthly boosters 83% were still alive compared to 60% survival in those dosed at monthly intervals and 50% in those with fortnightly dosing ± monthly boosts. The 0% survival rate of dogs receiving one dose or less represents the late stage cancers in these patients on enrollment in the trial. The results indicate that a more intense weekly vaccination schedule for four doses up front may improve response rates and outcomes.

The wide range of cancers treated by autologous vaccine varied from blood cancers (lymphoma) to various solid tumor types (mast cell tumor, osteosarcoma etc.). Although not a controlled study, suggestion of a possible positive effect of vaccination was seen for carcinomas, sarcomas and mast cell tumors. Interestingly, 4/5 subjects with B cell lymphomas that survived less than 3 months were all treated with a vaccine containing rhizavidin, whereas the other case with the stable disease at 9 months received only tumor lysate and Advax™. Given the overall negative trend in patients receiving rhizavidin in their vaccine formulation, it has been excluded from future studies. This highlights the value of treating canine patients with spontaneous tumors in a clinical setting before translating to human therapy.

The autologous vaccine containing Advax™ adjuvant proved to be a safe alternative with significant clinical benefits due to ease of administration. The vaccination schedule was well accepted by dog owners, and well tolerated by dogs with minimal side effects. Sedation or general anaesthesia was not required to administer the vaccine, and the pet could be returned to the owner quickly, which contrasts with chemotherapy and some radiation therapies where animal contact with the family must be restricted during the metabolism and excretion of the drug.

Lastly, the turn-around-time of several hours from tumor resection or biopsy to vaccine manufacture and subsequent administration significantly expedites treatment compared to other immunotherapy approaches.

## 5. Conclusions

In conclusion, these results demonstrate the feasibility of using Advax™-based autologous vaccines for the treatment of several types of naturally occurring canine cancers. The trial demonstrated safety and proof of concept, achieving positive clinical benefits in some patients including remission, static disease, and extended survival times in a clinical setting. Future controlled clinical trials with increased numbers of canine patients will be required to define clinical benefit.

## 6. Patents

Advax™ adjuvants are covered by patents owned by Vaxine Pty Ltd.

## Figures and Tables

**Table 1:** Details of 28 Canine patients treated with autologous vaccine. Abbreviations used: Diffuse Large B-cell Lymphoma (DLBCL), Mammary Carcinoma (MCA), Mast Cell Tumor (MCT), Squamous Cell Carcinoma (SCC). Stable Disease (SD), Complete Regression (CR), Lost to Follow Up (LTFU). Rhizavidin (RHIZ), Tumor Lysate (TL), * Never received vaccine.

Tumour Type	Stage/Grade/Location	Breed/Sex/Age	Sample size	Vaccine Composition	Vaccine doses	Survival time	Status	Medication/Observations
Diffuse large B-cell lymphoma (DLBCL)	stage IIIB	American Staffordshire Bullterrier (M5)	Biopsy 1.2 g	Advax/Rhiz/TL	2 (4 weeks apart)	10 weeks	Euthanased	Prednisolone 1 mg/kg BID
	stage V	Australian Kelpie X (FN 10)	Biopsy 1.4 g		2 (4 weeks apart)	4 weeks	Euthanased	Prednisolone 1 mg/kg BID
	stage V	Japanese Spitz X (MN 5)	Biopsy 1.4 g		4 (weekly)	6 weeks	Euthanased	Prednisolone 1 mg/kg BID
Mammary carinoma (MCA)		Bernese Mountain Dog (F10)	Resection 1.6 g		8 (4 weeks apart)	Still alive 8 months	New cancer	New, unrelated malignant tumour developed 7 months after starting vaccination
		Australian Cattle dog X (F13)	Resection 1.3 g		4 (4 weeks apart)	Still alive 4 months	New cancer	Histopathology pending on new cancer, might start vaccines again. New cancer 3 months after starting vaccination
Mast Cell tumour (MCT)	high grade (multiple tumours)	Golden Retriever (FN7)	Resection 1 g		5 (initially weekly × 4, then 4 weeks apart)	Still alive 2 months	CR	Had curative intent surgery for MCT recurrence and multiple new lesions.
	high grade	Rhodesian Ridgeback X (MN9)	Biopsy 0.6 g		9 (4 weeks apart)	Still alive 9 months	SD	Tumour no progression in size, no metastases
	high grade (multiple + mets in draining lymph nodes)	Staffordshire Bullterrier (M8)	Resection 1.1 g		1	3 weeks	Euthanased	Ranitidine 2 mg/kg BID, chlorpheniramine 8 mg BID
Non-epitheliotrophic T-cell lymphoma		Rottweiler (MN3)	Biopsy 0.6 g		11 (initially weekly, then 4 weeks apart)	Still alive 4 months	CR	No other medications, no lesions currently, 12 at beginning of vaccination
Epitheliotrophic T-cell lymphoma		Boxer (MN13)	Biopsy 0.5 g		9 (weekly, a bit random)	Still alive 3 months	SD	Some lesions have disappeared, but there are new ones also
		Labrador (MN11)	Biopsy 0.7 g		6 (weekly)	5 weeks	Euthanased	Went into complete remission with CCNU chemo but developed hepatopathy. Concurrent Prednisolone 0.75 mg/kg SID. PD
Squamous cell carcinoma (SCC)	oral	Shih Tzu X (MN12)	Biopsy 0.6 g		5 (initially weekly × 4, then 4 weeks apart)	Still alive 2 months	SD	Has undiagnosed masses in liver, adrenal and lungs
	oral	Cross breed (MN10)	Biopsy (0.1 g)		2	5 weeks	Euthanased	Unrelated causes
Soft tissue sarcoma (STS)	shoulder	Maltese Terrier (MN13)	Resection	Advax 2/Rhiz/TL	2 (2 weeks apart)	Unknown	LTFU	Owners uncontactable
Squamous cell carcinoma	left tonsil	Cross breed (MN8)	Multiple biopsies (0.36 g)		4 (2 × 2 weeks apart) then monthly	Unknown	LTFU	Owners uncontactable
Lymphoma (B cell)	multiple nodes	Terrier cross (MN11)	Biopsy (0.26 g)	Advax/TL	2 doses 2 weeks apart	2 months	Euthanased	Relapse
	multiple nodes	Groodle X (FN2.5)	Biopsy 0.4 g		4 × 1 weekly then monthly boosts	Still Alive 9 months	SD	Had prednisone initially
Mast Cell tumour	Low grade	Labrador (FN12)	Resection 7.27 g		> 4 doses—2 × 2 weekly then monthly	Still Alive 13 months	CR	No recurrance sice vaccination
	grade 3 neck	Beagle (FN10)	0.3 g Incomplete Resection		4 × 1 weekly then monthly boosts	Still Alive 9 months	SD	No tumour progression
Haemangiosarcoma	nasal	Staffordshire Bull terrier (MN9)	Debulk (0.6 g)		4 doses—2 × 2 weekly then Monthly	6 months	Euthanased	Aggressive disease
	spleenic	German Shepherd X (FN9)	Debulk > 1 g		4 × 1 weekly then monthly boosts	Still Alive 7 months	SD	Stable disease exceeding expectations
Squamous cell Carcinoma	nasal	Alaskan Malamute (MN9)	Debulk 1.6 g		> 4 doses—2 × 2 weekly then monthly	Still Alive 12 months	SD	Still residual disease
Sarcoma	grade 1 nose	Australian Terrier (MN12)	Debulk 1 g		> 4 doses—2 × 2 weekly then monthly	Still Alive 12 months	SD	Radiotherapy as well (stable disease)
Osteosarcoma	grade 3	Pyrenean Mt Dog (MN12)	0.5 g Amputation		1 dose	2 weeks	Euthanased	Complications from Surgery
	grade 3 (MI 15)	Labrador (MN10)	Amputation 0.1 g biopsy		4 × 1 weekly then monthly boosts	Still Alive 7 months	SD	Sample depleted for vaccine production
Carcinoma	primary (right hind leg) + lymph node (mets)	Springer Spaniel (MN11)	0.6 g Resect		4 × 1 weekly then monthly boosts	Still Alive 9 months	SD	Stable even with metastatic disease
Hepatocelluar Carcinoma	liver + lipoma on outer sections	Border Collie X (MN12)	Biopsy < 0.1 g		4 × 1 weekly then monthly boosts	Still Alive 9 months	SD	Sample depleted for vaccine production
